# Amyloid-precursor Like Proteins APLP1 and APLP2 Are Dispensable for Normal Development of the Neonatal Respiratory Network

**DOI:** 10.3389/fnmol.2017.00189

**Published:** 2017-06-22

**Authors:** Kang Han, Ulrike C. Müller, Swen Hülsmann

**Affiliations:** ^1^Institute of Pharmacy and Molecular Biotechnology, Heidelberg UniversityHeidelberg, Germany; ^2^Klinik für Anästhesiologie, Universitätsmedizin GöttingenGöttingen, Germany; ^3^Center for Nanoscale Microscopy and Molecular Physiology of the Brain (CNMPB)Göttingen, Germany

**Keywords:** amyloid-precursor like proteins, pre Bötzinger complex, medullary slice

## Abstract

Recent studies using animal models indicated that the members of the amyloid precursor protein (APP) gene family are important for the formation, maintenance, and plasticity of synapses. Despite this, the specific role of the APP homologs APLP1 and APLP2 within the CNS and PNS is still poorly understood. In contrast to the subtle phenotypes of single mutants, double knockout mice (DKO) lacking APP/APLP2 or APLP1/APLP2 die within the first day after birth. Whereas APP/APLP2-DKO mice show severe deficits of neuromuscular morphology and transmission, the underlying cause of lethality of APLP1/APLP2-DKO mice remains unclear. Since expression of both proteins was confirmed by *in situ* hybridization, we aimed to test the role of APLP1/APLP2 in the formation and maintenance of synapses in the brainstem, and assessed a potential dysfunction of the most vital central neuronal network in APLP1/APLP2-DKO mice by analyzing the respiratory network of the medulla. We performed *in vivo* unrestrained whole body plethysmography in newborn APLP1/APLP2-DKO mice at postnatal day zero. Additionally, we directly tested the activity of the respiratory network in an acute slice preparation that includes the pre-Bötzinger complex. In both sets of experiments, no significant differences were detected regarding respiratory rate and cycle variability, strongly arguing against central respiratory problems as the primary cause of death of APLP1/APLP2-DKO mice. Thus, we conclude that APLP1 and APLP2 are dispensable for the development of the network and the generation of a normal breathing rhythm.

## Introduction

Amyloid precursor-like proteins 1 (APLP1) and 2 (APLP2) are type I transmembrane proteins belonging to the evolutionary conserved amyloid precursor protein (APP) gene family (Coulson et al., 2000; Muller et al., 2017). APP has been intensely studied with regard to Alzheimer’s disease (AD) pathogenesis, as proteolytic processing of APP gives rise to the Aβ peptide that is deposited in extracellular plaques in the brains of Alzheimer patients (Selkoe and Hardy, 2016). Although the Aβ region is unique for APP the two APLPs share with APP an overall similar structural organization and several conserved domains (Fox et al., 2007). Moreover, they are processed by the same set of α-, β- and γ- secretases yielding a complex array of proteolytic fragments(Slunt et al., 1994; Scheinfeld et al., 2002; Eggert et al., 2004; Endres et al., 2005; Kuhn et al., 2016). During development and in adult rodents APP and APLP2 are ubiquitously expressed in a largely overlapping pattern in many tissues with particularly high expression in the nervous system (brain, spinal cord, retina, ganglia) including the neuromuscular junction (NMJ) (Slunt et al., 1994; Lorent et al., 1995; Sarasa et al., 2000; Wang et al., 2005; Caldwell et al., 2013; Klevanski et al., 2014; see also footnote^1^).

In contrast, APLP1 is specifically expressed in neurons (Lorent et al., 1995). APP and APLPs are found in the somatodendritic and axonal compartment (Schilling et al., 2017) and have been localized to the presynaptic active zone (Kim et al., 1995; Walsh et al., 2007; Lassek et al., 2013). APP family proteins can form homotypic and heterotypic dimers and have been implicated in transcellular and synaptic adhesion *in vitro* and *in vivo* at the NMJ (Soba et al., 2005; Wang et al., 2009; Baumkotter et al., 2014). Their physiological functions have been studied using knockout (KO) mice lacking either individual family members or in all possible combinations of DKO and triple KO mice (Aydin et al., 2012; Mockett et al., 2017; Muller et al., 2017). All three single KO mice exhibit rather subtle phenotypes and display no apparent alterations in brain morphology (Zheng et al., 1995; von Koch et al., 1997; Magara et al., 1999; Heber et al., 2000). APP-KO mice, which have been most thoroughly studied, show reduced brain and bodyweight (15–20%), reduced grip strength and locomotor activity and increased susceptibility to brain injury (Zheng et al., 1995; Ring et al., 2007; Hefter et al., 2016; Plummer et al., 2016). Reduced theta-gamma coupling in APP-deficient mice (Zhang et al., 2016) points toward alterations in the connectivity of central networks (Zhang et al., 2016), but only aged (12-month-old) APP-KO mice exhibit reduced spine density in cortex and hippocampus, impairments in long term potentiation (LTP) at CA3/CA1 synapses of the hippocampus and impairments in spatial learning (Dawson et al., 1999; Seabrook et al., 1999; Ring et al., 2007; Lee et al., 2010; Tyan et al., 2012). Apart from subtle retinal abnormalities (Dinet et al., 2016) APLP2-KO mice show a wild type like phenotype with normal spine density and synaptic plasticity even at old age (von Koch et al., 1997; Heber et al., 2000; Weyer et al., 2011; Midthune et al., 2012). APLP1-KO have been studied in much less detail compared to APP and APLP2 deficient mice. Similar to APP-KO mice, APLP1-KO mice show reduced body weight but normal locomotor activity and grip strength (Heber et al., 2000). Electrophysiological analysis of perforant path-granule cell synapses of the dentate gyrus revealed decreased network inhibition but no alterations in LTP in APLP1-KO mice (Vnencak et al., 2015). In line with this, morphological analysis of CA1 neurons in organotypic hippocampal cultures revealed normal spine density and dendritic branching (Weyer et al., 2014). However, recent analysis of aged (1-year-old) APLP1-KO mice showed reduced spine density and frequency of miniature excitatory synaptic currents in the hippocampus pointing toward compensatory mechanism that may fail with aging (Schilling et al., 2017).

Genetic evidence indicates that the above mentioned- rather minor- phenotypes of single KOs are likely due to functional compensation within the gene family. APP/APLP2 DKO mice, APLP1/APLP2-DKO and triple KO mice die within the 1st day after birth (von Koch et al., 1997; Heber et al., 2000; Herms et al., 2004). Interestingly, APP/APLP1-DKO mice proved viable, indicating that APLP2 has unique properties that are required when either APP or APLP1 are lacking (Heber et al., 2000). Together these data suggest that APP family proteins can serve overlapping functions in tissue in which they are co-expressed (Muller et al., 2017).

Indeed, recent data suggest a functional compensation between APP and APLP2 in the CNS. Conditional, forebrain-specific APP/APLP2-DKO mice exhibited reduced spine density and branching of hippocampal neurons, impaired synaptic plasticity and pronounced impairments in hippocampus dependent behavior that were found already in young adult mice (Hick et al., 2015). Despite this, the specific role of the APP homologs APLP1 and APLP2 within the CNS and PNS is still poorly understood. Conditional APLP1/APLP2-DKO mice have not been generated so far, which precludes detailed analysis of neuronal network functions of APLP1 and APLP2 in the adult brain. However, analysis of the networks in the brainstem, that are already developed at birth, especially the respiratory network (Feldman et al., 2012; Dick et al., 2015) is possible and thus allowed us to gather information about basic synaptic connectivity in otherwise lethal APLP1/APLP2-DKO mice. Analysis of this vital network appears even more reasonable, since the morphology of the NMJ appears normal in APLP1/APLP2-DKO mice. Unlike, APP/APLP2-DKO mice, which show a strongly altered morphology of NMJs at the diaphragm and severely impaired neurotransmission (Wang et al., 2005, 2007, 2009; Caldwell et al., 2013; Klevanski et al., 2014), APLP1/APLP2-DKO mice showed normal endplate patterning and only very subtle morphological abnormalities of individual synapses, which strikingly contrasts with the highly penetrant lethality of these mutants [less than 0.5% of APLP1/APLP2-DKO pups survive up to weaning (Klevanski et al., 2014)].

Therefore, we analyzed the respiratory network of newborn mice using whole body plethysmography and direct electrophysiological recordings from brain stem slices. However, we did not observe significant differences between APLP1/APLP2-DKO pups and littermates, suggesting that APLP1 and 2 are not essential for respiratory network formation and function.

## Materials and Methods

### Animal Ethics

Experiments were conducted in accordance with the guidelines of the German Physiological Society, the European Communities Council Directive and the law of Federal Republic of Germany. Breeding of perinatally lethal APLP1/APLP2-DKO mice (Heber et al., 2000) to obtain tissue samples and brain slices has been approved by the Regierungspräsidium Karlsruhe (35-9185.81/G-82/14).

### *In Situ* Hybridization

The gene sequence of the APLP1 probe corresponds to nucleotides 1431–1940 of the murine APLP1 mRNA as previously described (Vnencak et al., 2015). The gene sequence of the APLP2 probe corresponds to nucleotides 1554–2100 of APLP2 mRNA (GenBank accession number NM_001102456.1, for full sequence see **Table 1**). DIG labeled RNA probes were *in vitro* transcribed using the Roche DIG RNA labeling kit (SP6/T7) and purified using RNAse free ChromaSpin 100 columns (Clontech). The quantity of labeled and purified probe was estimated by Dot blot as described in the DIG RNA labeling kit manual. Brains of P0 mice were fixed in 4% PFA/DEPC-PBS over night at 4°C followed by three washes in PBS (3 min each), and dehydrated through an ascending sucrose series (10%; 15%; 30%) diluted in DEPC-PBS. OCT (Tissue –Tek) embedded brains were frozen on dry ice, and finally stored at -80°C. Brains were cut on a cryostat (Zeiss Hyrax C50) at a thickness of 30 μm. Brain sections were post-fixed with 4% PFA/DEPC-PBS for 20 min, and treated with Proteinase K (10 μg/ml in 20 mM Tris/HCl, 1mM EDTA, pH 7.2) for 10 min, washed in 3 times of DEPC-PBS (10 min each), and then put horizontally into a chamber humidified with 50% formamide/4× SSC. Each slide was covered with 100 μl hybridization buffer plus 400 pg labeled probe. Hybridization was carried out over night at 56°C in the tightly sealed humidified chamber. On the next day, coverslips were floated off in 5× SSC to wash away excess probe (10 min). Stringency washes were 20 min in 5× SSC for 3 times and 40 min in 0.5× SSC/20% formamide at 60°C in a water bath. Slides were cooled down to RT in 0.5× SSC/20% formamide at RT, and washed 15 min in NTE (0.5 M NaCl, 10 mM Tris pH 7.0, 5 mM EDTA) at 37°C, treated with 10 μg/ml RNase A/NTE for 30 min at 37°C, followed by a 15 min wash in NTE. After a further 40 min wash in 0.5× SSC/20% formamide at 60°C slides were equilibrated in P1 DIG (100 mM Tris/HCl; 150 mM NaCl) for 10 min and afterwards blocked in blocking solution (P1DIG + 0.5% BSA + 1% Blocking reagent, Roche) for 30 min. Brain slices were encircled with PAP PEN, anti-DIG-AP antibody (Roche) was diluted 1:500 in blocking solution, and 80 μl were pipetted onto every brain slice. Antibody incubation was overnight at 4°C in a humidified chamber. The next day, all slides were washed twice for 15 min in P1 DIG and then equilibrated in P3 DIG (100 mM Tris/HCl; 100 mM NaCl; 50 mM MgCl2, pH 9.5) for 2 min. 80 μl substrate solution for alkaline phosphatase (NBT/BCIP stock solution, diluted 1:50 in P3 DIG) were pipetted onto each brain slice, incubation was overnight at RT until color development (due to the formation of the insoluble, violet NBT diformazan) was sufficient. Slides were then washed in PBS-T, fixed for 10 min in 4% PFA in PBS, washed in P4 DIG (10 mM Tris/HCl; 1 mM EDTA, pH 8.0) for 10 min, air dried for 2 h and finally mounted in Mowiol.

**Table 1:** Amyloid precursor-like proteins 2 probe *in situ* hybridization.

APLP2	
mRNA	Nucleotides 1554-2100
Species	Mus musculus
GenBank	NM_001102456.1
Sequence	GCTCGAAATT AACCCTCACT AAAGGGAACA AAAGCTGGAG CTCCACCGCG GTGGCGGCCG CTCTAGACTC TTCTGTACAA AGTTCCTTAT GTTGCTCAAG AAATTCAAGA GGAAATTGAT GAGCTCCTTC AGGAACAGCG AGCGGATATG GACCAATTTA CCTCCTCCAT CTCAGAGAAC CCTGTGGATG TCCGGGTGAG CTCTGAGGAG AGTGAGGAGA TCCCGCCGTT CCACCCTCTC CATCCCTTCC CATCCTTGTC TGAGAATGAA GACACTCAGC CGGAGTTGTA CCACCCAATG AAAAAAGGCT CTGGAATGGC AGAACAAGAC GGGGGACTGA TTGGTGCAGA AGAAAAAGTG ATTAACAGCA AGAATAAAAT GGATGAAAAT ATGGTCATTG ACGAGACTCT GGATGTTAAG GAAATGATTT TCAATGCTGA GAGAGTTGGA GGCCTTGAGG AAGAGCCGGA ATCGGTGGGA CCTTTAAGGG AGGATTTCAG TTTGAGCAGC AATGCCCTTA TTGGCTTGCT GGTTATCGCA GTGGCCATTG CTACGGTCAT CGTTATCAGC CTGGTGATGC TGAGGAAGAG GCAGTACGGC ACCATCAGCC ACGGCTCGAG GGGGGGCCCG GTACCCAATT CGCCCTATAG TGAGTCGTAT TA

### Microscopy and Image Processing

**FIGURE 1:**
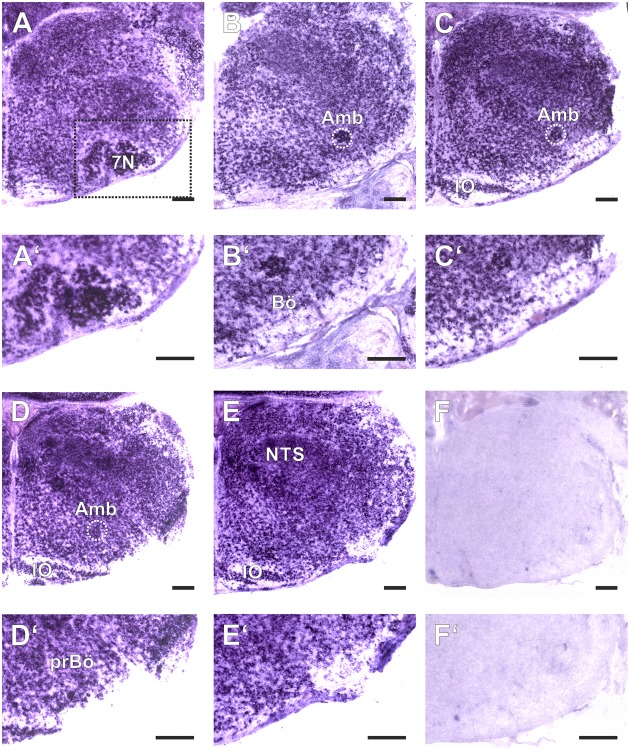
Amyloid precursor-like proteins 1 *in situ* hybridization (ISH) in the medulla oblongata of a P0 mouse using a DIG-labeled antisense APLP1 probe. **(A–E)** show hemi sections of the brainstem in rostro-caudal direction form the level of the facial nucleus (7N; **A**) to the level the lateral reticular nucleus (LRt; **E**); as a negative control we used a section from an APLP1-KO mouse **(F)** High magnification images of the ventral lateral medulla and ventral respiratory column (VRC) are shown in **(A′–F′)**. The region of the pre Bötzinger Complex (prBö) is shown in panels **(D,D′)**. Scale bars correspond to 100 μm. IO = inferior olive, Amb = nucleus ambiguous (encircled). Note the violet staining due to the alkaline phosphates mediated formation of the NBT diformazan (see Materials and Methods).

**FIGURE 2:**
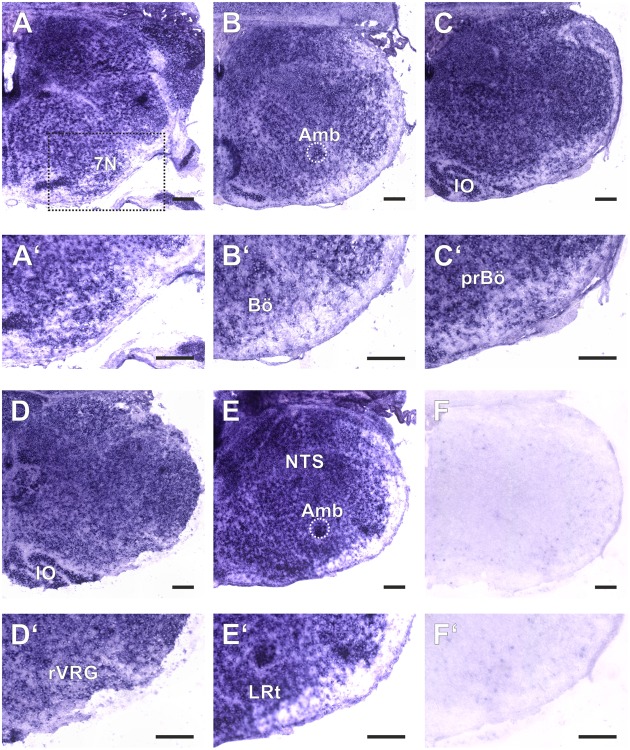
Amyloid precursor-like proteins 2 ISH in the medulla oblongata of a P0 mouse using a DIG-labeled antisense APLP2 probe. **(A–E)** show hemi sections of the brainstem in rostro-caudal direction form the level of the facial nucleus (7N; **A**) to the level the lateral reticular nucleus (LRt; **E**); as a negative control we used a section from an APLP2-KO mouse **(F)**. High magnification images of the ventral lateral medulla and VRC are shown in **(A′–F′)**. The region of the pre Bötzinger Complex (prBö) is shown in panels **(C,C′)**. Scale bars correspond to 100 μm. IO = inferior olive, Amb = nucleus ambiguous (encircled). Note the violet staining due to the alkaline phosphates mediated formation of the NBT diformazan (see methods).

Images from ISH were taken with a Keyence BZ-9000 microscope, using a 20× objective. Tiled images were automatically generated from 20 high resolution images (1340×1024 pixel CCD sensor) using the Image Joint Function of BZ-II Analyzer software (Keyence). Scaling was performed for data reduction leading to final images (1760 × 1805 Pixel). Final images were exported to tif-format (8bit-RGB) and composed to final figures in a graphic program (CorelDraw). The “Paxinos Atlas of the Developing Mouse Brain” (Paxinos, 2007) was used to identify the brainstem structures depicted in **Figures 1**, **2**.

### Unrestrained Whole-Body Plethysmography

Resting ventilation was measured using unrestrained whole-body plethysmography (Bartlett and Tenney, 1970) adapted for use with neonatal animals: Individual animals were placed in a chamber (5–10 ml) that was connected to one side of a differential pressure transducer (model DP103-14, Validyne Engineering, Northridge, CA, United States). The chamber communicated with atmospheric pressure through a slow leak (27 gauge hypodermic needle) to minimize pressure differences between the chambers because of fluctuations in atmospheric pressure during measurements. The analog signal from the transducer was demodulated (model CD-15 carrier demodulator, Validyne Engineering), amplified, filtered and recorded on thermal chart recorder. Additionally, data were digitized (≥ 1kHz) using an interface (ITC-16; HEKA, Lambrecht, Germany) and then captured and stored to disk by Apple computer (Acquire, Bruxton Corporation, Seattle, WA, United States or Axograph 4.0, Axon Instruments, Foster City, CA, United States). The ventilatory pattern was recorded from each animal for 2–3 min on postnatal day 1 during the first 8 h after birth. Measurements were performed at room temperature (Gomeza et al., 2003), however, the brief time away from the nest would have minimal effect on body temperature. Moreover, WT and KO animals were treated in exactly the same way, since the experimenter was blind to the genotypes. To remove drift from the recordings a digital filtering was performed (Band pass 0.5–20 Hz; LabChart 8).

### Slice Preparation

Immediately after the plethysmography, mice were anesthetized with ether. The brain and upper cervical spinal cord were isolated in ice-cold artificial cerebrospinal fluid (aCSF), which was saturated with carbogen (95% O_2_–5% CO_2_). The brainstem was isolated and glued with cyano-acrylate to an agar block with its rostral end directed upward. Brainstem slicing was started from the rostral end with the neuroaxis inclined by 20° to the plane of the blade. This configuration preserved most projections from the pre-Bötzinger complex to the nucleus of hypoglossus and left the hypoglossal (XII) rootlets intact (Ramirez et al., 1996). A 650–750 μm thick cut was made to obtain one slice containing the functionally intact respiratory center (Gomeza et al., 2003). This preparation includes the pre-Bötzinger complex (preBötC), a region which is essential for the generation of the respiratory rhythm (Smith et al., 1991). The slice was transferred to a recording chamber and stabilized by a platinum frame with nylon fibers. The XII rootlet was drawn into a suction electrode, or alternatively an extracellular electrode filled with aCSF was placed in the hypoglossal nucleus. The concentration of extracellular K^+^ in aCSF saturated with carbogen at 30°C was increase to 8 mM to maintain respiratory network activity. Extracellular neuronal activity was amplified 5’000–10’000 times, band-pass (0.25–1.5 kHz) filtered, rectified, and integrated (Paynter filter with a time constant of 50–100 ms) (Hulsmann et al., 2000). Hypoglossal activity, which corresponds to the inspiratory phase of the respiratory cycle (Smith et al., 1990) can be used as an index of the central respiratory rhythm (Smith et al., 1991). Rootlet discharges and their integrals were digitized at 5 kHz using an interface (ITC-16; Instrutech Corp., Great Neck, NY, United States) and Axograph 4.0 software (Axon Instruments, Inc., Foster City, CA, United States). Data were stored on hard disk for off-line analysis. Burst interval and amplitude of the integrated rootlet signal were measured with Axograph 4.0 software. Burst frequency was calculated as the reciprocal of the mean inter-burst interval. The coefficient of variation (CV) of the amplitude of the integrated burst was used as an additional parameter of the overall network activity. Results are expressed as means ± SE. One Way Analysis of Variance (ANOVA) tests were used to determine the significance of changes using Sigma Plot software (SPSS Inc., Chicago, IL, United States). Results were considered significant if *P* < 0.05. The aCSF contained (in mM) 118 NaCl, 3 KCl, 1.5 CaCl_2_, 1 MgCl_2_, 1NaH_2_P04, 25 NaHCO_3_ and 30 D-glucose (pH = 7.4, 310 mosmol/l) at a temperature of 30°C. Substances were purchased from Sigma (Deisenhofen, Germany) unless otherwise indicated.

### Data Handling and Statistical Analysis

Figures were assembled using the graphic program CorelDraw. Statistical analysis was performed with SigmaPlot software using One Way Analysis of Variance (ANOVA) tests. Significance was assumed if *p* < 0.05.

## Results

In this paper we addressed how a deficiency of both APLP1 and APLP2 affects the respiratory network of newborn mice and ask the question whether APLP1/APLP2 DKO mice die because of a functional defect of the respiratory network in the medulla oblongata. To this end, we first assessed the level of APLP1 and APLP2 expression in the medulla. Second, we measured breathing using whole body plethysmography to test if the animals are able to ventilate. Last, we tested the neuronal activity of the kernel of the respiratory network, the pre-Bötzinger complex, which allows to test if the neuronal interaction in the network is grossly intact (Gomeza et al., 2003; Rahman et al., 2015).

### Expression of APLP Proteins in the Respiratory Network of the Medulla

As a baseline for further experiments we studied the expression pattern of APLP1 and 2 in the medulla of newborn wild type mice. As reliable antibodies that work in immunohistochemistry are not available for APLPs, we assessed mRNA expression by *in situ* hybridization (ISH). Brain sections from APLP1-KO and APLP2-KO mice were processed in parallel and served as a negative control. We found substantial and largely overlapping expression of both APLPs in the ventrolateral medulla. High level expression of APLP1 was detected in motor nuclei of the medulla especially in the facial nucleus, at the nucleus ambiguus and in the inferior olive (**Figure 1**). Along the ventral respiratory column (VRC) substantial expression is also found in the Bötzinger Complex (BötC), the Pre-Bötzinger Complex (preBötC) and in the rostral Ventral respiratory group (rVRG). Additionally, neurons in the nucleus of the solitary tract, which is part of the dorsal respiratory column, also expressed APLP1. A similar expression pattern was observed for APLP2 (**Figure 2**), however, the expression in the facial nucleus and inferior olive was less prominent as compared to the neighboring structures. Similar to APLP1 substantial expression of APLP2 mRNA was found in the VRC (including BötC, preBötC, and rVRG). In contrast, only a weak background staining was observed in APLP1-KO and APLP2-KO sections.

### Breathing of Newborn Mice

In total we obtained 4 litters with 42 offspring (APLP1^+/+^APLP2^-/-^ (APLP1-WT): *n* = 7; APLP1^+/-^APLP2^-/-^ (APLP1-heterozygous): *n* = 18; APLP1^-/-^APLP2^-/-^ (DKO): *n* = 17). Among these, we found 4 dead offspring in the cage from which 3 newborns were genotyped as DKO and one as APLP1-heterozygous.

**FIGURE 3:**
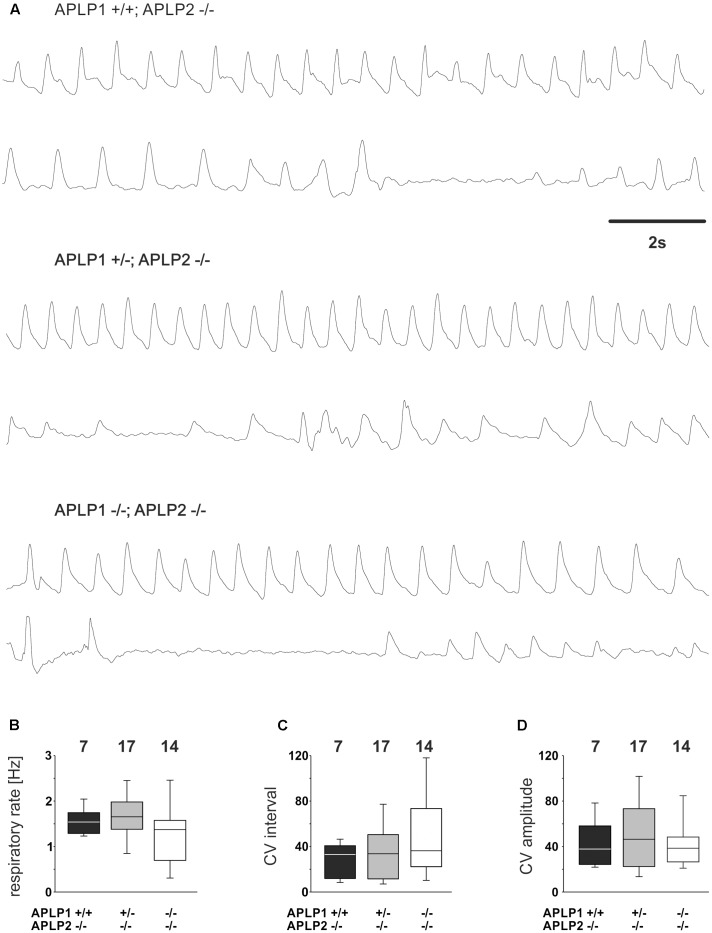
Breathing of APLP1, APLP2 double knock-out mice recorded by whole-body plethysmography. **(A)** Example traces from recordings for three different genotypes. **(B–D)** Quantification of statistical evaluation the respiratory rate **(B)**, and variability of the breathing. The Coefficient of Variation (CV) of the respiratory interval is shown in **(C)**, the CV of the amplitude in **(D)**. In **(B–D)**, the lower boundary of the box is the 25th percentile, lines within a box represents the median, the higher boundary of the box provides 75th percentile. Error bars indicate the 10th and 90th percentiles, respectively. Number of animal analyzed are given on top of the boxes.

Having established that APLP1 and APLP2 are co-expressed in the respiratory brain stem we now aimed to identify a potential central respiratory insufficiency that could be causal for the early death of DKO mice at postnatal day zero (Heber et al., 2000). Therefore, breathing of the neonates was measured (within the first 8 h after birth) with unrestrained whole-body plethysmography. From 14 analyzed APLP1^-/-^APLP2^-/-^ mice, two showed extremely long apneic intervals (≥ 10 s; not shown). However, shorter apneas (2–10 s) were also found in the other genotypes (APLP1^+/+^APLP2^-/-^ mice = two animals; APLP1^+/-^APLP2^-/-^ mice = 7 animals; APLP1^-/-^APLP2^-/-^ mice = 4 animals; **Figure 3**). The average duration of the longest apnea (or cycle interval) detected during the recordings were not significantly different between APLP1^+/+^APLP2^-/-^ mice (1.9 ± 0.5 s mean ± SEM; median: 1.5 s), APLP1^+/-^APLP2^-/-^ mice (1.7 ± 0.3 s; median: 1.5 s) and APLP1^-/-^APLP2^-/-^ mice (4.0 ± 1.1 s; median: 1.6 s; ANOVA on Ranks; *p* = 0.578).

Further quantitative analysis of the breathing rate of surviving mice was, however, not different between genotypes (**Figure 3A**). APLP1^+/+^APLP2^-/-^ had on average a rate of 1.58 ± 0.11 Hz (mean ± SEM). APLP1^+/-^APLP2^-/-^ mice (*n* = 7) ventilated with an average rate of 1.64 ± 1.3 Hz and APLP1^-/-^APLP2^-/-^ mice with a rate of 1.29 ± 0.18 Hz (*p* = 0.206). There was a trend toward a higher variability of the respiratory cycle length in APLP1^-/-^APLP2^-/-^ mice. Although the variability of the respiratory cycle tended to be larger in DKO mice, the difference of the coefficient of variation (CV) of the inter burst interval between APLP1^+/+^, APLP2^-/-^ (29.4 ± 5.4), APLP1^+/-^, APLP2^-/-^ (34.3 ± 6.2) and DKO (50.0 ± 10.3) remained non-significant (*p* = 0.489). Similarly, also no significant differences for the CV of the amplitude was detected between APLP1^+/+^APLP2^-/-^ (43.2 ± 7.5), APLP1^+/-^APLP2^-/-^ (49.7 ± 7.4) and APLP1^-/-^APLP2^-/-^ (41.8 ± 5.8). These data show that APLP1^-/-^APLP2^-/-^ DKO mice are able to breath at birth. Thus, a primary problem resulting from a defect in the development of the respiratory network appears unlikely.

### Analysis of Respiratory Network Function *In Situ*

**FIGURE 4:**
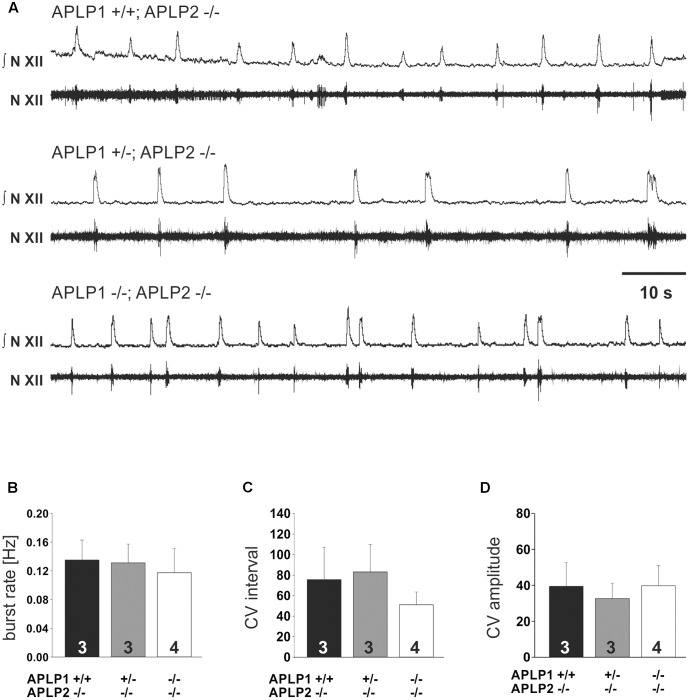
*In vitro* respiration as recorded by hypoglossal neuronal activity in isolated medullary slice containing the pre-Bötzinger complex. **(A)** Example traces from recordings for three different genotypes. The upper traces (N.XII) represent the integrated signal of the original recording from hypoglossal nucleus (lower trace; N. XII). **(B–D)** Basic statistical summary of the burst rate **(B)**, and variability **(C,D)**. The CV of the inter burst interval is shown in **(C)**, the CV of the amplitude in **(D)**. Values are depicted as average ± SEM. Number of animal/slices analyzed are given inside the bars.

To substantiate our interpretation and to investigate the brainstem respiratory network independent of arousal or other central neuronal factors that might influence breathing we also analyzed central respiratory network activity in the rhythmic slice preparation from the caudal brainstem including the pre-Bötzinger complex. We recorded rhythmic hypoglossal motoneuron pool discharges, which are known to occur in synchrony with periodic bursts of neurons in the pre-Bötzinger complex. The frequency and the CV of the burst discharge of hypoglossal motoneuron pool was very similar in all three groups of mice (**Table 2** and **Figure 4**). Taken together, these findings suggest that the anatomy and the connectome of neonatal respiratory network is intact and that the mice do not die from a failure of respiratory rhythm generation.

**Table 2:** Summary of the activity of the respiratory network *in vitro* (N. XII recordings).

	APLP1^+/+^ APLP2^-/-^	(*n* = 3)	APLP1^+/-^	APLP2^-/-^	(*n* = 3)	APLP1^-/-^	, APLP2^-/-^	(*n* = 4)
Burst rate [Hz]	0.14 ± 0.03	0.13 ± 0.03	0.12 + 0.04
CV interval [%]	75.6 ± 31.7	83.1 ± 26.8	51.7 ± 12.3
CV amplitude [%]	39.5 ± 13.2	32.7 ± 8.5	29.7 ± 7.3

## Discussion

*In situ* expression analysis demonstrated that both APLP mRNAs are expressed in brain stem areas important for respiratory function. This is well in agreement with previous studies that reported expression of APLP1 in the spinal cord and brain stem of E18.5 wild type mice^2^. However, the resolution was insufficient to unequivocally demonstrate expression, e.g., in nuclei involved in the control of respiratory rhythm generation. Expression of APLP2 mRNA had only been shown in spinal cord at E15, but not assessed in brain stem or close to birth (Lorent et al., 1995). Thus, our findings that both APLPs show an overlapping expression pattern in the medulla were in line with our initial hypothesis that a combined lack of both APLPs might disturb breathing. Our subsequent findings do, however, not support this notion.

Since breathing frequency is not different in APLP1/APLP2-DKO mice in comparison to the APLP1 positive mutants, we can argue that a developmental defect in the respiratory network is unlikely to be responsible for the early postnatal death of the double knock out mice. Unlike in mice that have been shown to die from a central respiratory failure, presenting with either no breathing movements (Rahman et al., 2015) or extremely long apneas (Blanchi et al., 2003; Gomeza et al., 2003) immediately after birth, breathing of APLP1/APLP2-DKO mice was neither slower nor strikingly more irregular than in viable littermates that were heterozygous or wild type for APLP1. Moreover, the observation of a normal breathing rhythm is in line with a normal development of diaphragm innervation as demonstrated by a normal endplate distribution and branching pattern of the phrenic nerve (Klevanski et al., 2014).

Irregular breathing patterns with periods of apnea (**Figure 3**) are not uncommon in wild type mice at postnatal day zero (Robinson et al., 2000). Thus, longer apneas, which were observed in 2 APLP1/APLP2-DKO pubs, should be considered secondary to a yet unknown cause of deterioration of the mouse and might therefore reflect the agony of the dying animal. Further assessment of the respiratory network properties in respiratory rhythmic slice preparation containing the pre-Bötzinger Complex supported this notion. Frequency of inspiratory burst as recorded from the hypoglossal motoneurons in APLP1/APLP2-DKO mice was indistinguishable from APLP2 single knock out and heterozygous APLP1^+/-^APLP2^-/-^ littermates (**Figure 4**). Thus, there is no evidence for a general disturbance of the respiratory network as an elementary cause of the death. Moreover, the persistence of respiratory activity argues against a substantial disturbance of synaptic interaction in the respiratory network, which is in line with the absence of obvious ultrastructural changes of brain stem synapses in APLP1/APLP2-DKO mice (Heber et al., 2000) although a quantitative analysis has not been performed. In this regard it is interesting that for APP/APLP2-DKO mice reduced synaptic vesicle density and active zone size was reported for submandibular ganglion synapses (Yang et al., 2005). Nevertheless it is unlikely that APP family proteins are essential for basal synaptic transmission of CNS neurons, as excitatory neurons derived *in vitro* from triple KO embryonic stem cells showed normal spontaneous mEPSC frequencies and amplitudes (Bergmans et al., 2010) consistent with unaltered basal synaptic transmission as demonstrated by normal input/output strength recorded at the CA3/CA1 pathway in forebrain-specific APP/APLP2-DKO mice (Hick et al., 2015). More recent data point toward a regulatory role of APP family proteins to facilitate neurotransmitter release, as proteins of the release machinery including Munc18 and synaptotagmins have been found to interact with APP and the APLPs (Weyer et al., 2011; Fanutza et al., 2015).

All three APP family proteins have been shown to interact with NMDA receptors and enhance their cell surface expression in transfected cells (Cousins et al., 2009, 2015). From this one might expect synaptic alterations in the forebrain and/or brain stem of APLP1/APLP2-DKO mice. Unfortunately, such data are currently unavailable and will await the generation conditional APLP1/APLP2-DKO mice. We also cannot rule out at present, whether APP may compensate for the loss of APLPs in the brain stem, although it is not sufficient to confer postnatal survival. With respect to a potential alteration of NMDA-receptor expression in APLP1/APLP2 KO mice, an obvious similarity to the phenotype of NMDAR1-deficient mice needs to be pointed out (Forrest et al., 1994). NMDAR1-deficent newborn mice also develop a lethal phenotype with prolonged apneas and death, but the respiratory activity in medullary slice preparation, is indistinguishable from controls (Funk et al., 1997).

In summary, in the presence of APP, amyloid precursor-like proteins APLP1 and APLP2 are dispensable for a normal organization of the respiratory network during embryonic development. Thus, alteration of synaptic function in other brain areas like the arousal system, or even non-neuronal and metabolic effects, such as hypoglycaemia previously shown for APP/APLP2-DKO mice (Needham et al., 2008), might be a potential cause of neonatal death. Finally, our findings may also warrant to re-examine APLP1 and APLP2 mediated functions at the NMJ. Although morphological alterations in APLP1/APLP2-DKO were very subtle this does not preclude functional defects, e.g., for transmitter release as previously detected specifically at neuromuscular synapses of APP/APLP2-DKO mice (Wang et al., 2005, 2009).

## Author Contributions

SH and UM designed the study. SH and KH performed experiments. SH, KH, and UM wrote the manuscript.

## Conflict of Interest Statement

The authors declare that the research was conducted in the absence of any commercial or financial relationships that could be construed as a potential conflict of interest.

## Footnotes

1 http://developingmouse.brain-map.org

2 http://developingmouse.brain-map.org
